# Overview on wearable sensors for the management of Parkinson’s disease

**DOI:** 10.1038/s41531-023-00585-y

**Published:** 2023-11-02

**Authors:** Caroline Moreau, Tiphaine Rouaud, David Grabli, Isabelle Benatru, Philippe Remy, Ana-Raquel Marques, Sophie Drapier, Louise-Laure Mariani, Emmanuel Roze, David Devos, Gwendoline Dupont, Matthieu Bereau, Margherita Fabbri

**Affiliations:** 1 Department of Neurology, Parkinson’s disease expert Center, Lille University, INSERM UMRS_1172, University Hospital Center, Lille, France; 2 The French Ns-Park Network, Paris, France; 3 CHU Nantes, Centre Expert Parkinson, Department of Neurology, Nantes, F-44093 France; 4 Assistance Publique Hôpitaux de Paris, Department of Neurology, CIC Neurosciences, Pitié-Salpêtrière Hospital, Sorbonne University, Paris, France; 5 Sorbonne University, Paris Brain Institute - ICM, Inserm, CNRS, Paris, France; 6 Department of Neurology, University Hospital of Poitiers, Poitiers, France; 7 INSERM, CHU de Poitiers, University of Poitiers, Centre d’Investigation Clinique CIC1402, Poitiers, France; 8 Centre Expert Parkinson, NS-Park/FCRIN Network, CHU Henri Mondor, AP-HP, Equipe NPI, IMRB, INSERM et Faculté de Santé UPE-C, Créteil, FranceService de neurologie, hôpital Henri-Mondor, AP-HP, Créteil, France; 9 Université Clermont Auvergne, CNRS, Clermont Auvergne INP, Institut Pascal, Clermont-Ferrand University Hospital, Neurology department, Clermont-Ferrand, France; 10 Pontchaillou University Hospital, Department of Neurology, CIC INSERM 1414, Rennes, France; 11 Parkinson’s Disease Centre of Excellence, Department of Medical Pharmacology, Univ. Lille, INSERM; CHU Lille, U1172 - Degenerative & Vascular Cognitive Disorders, LICEND, NS-Park Network, F-59000 Lille, France; 12 Centre hospitalier universitaire François Mitterrand, Département de Neurologie, Université de Bourgogne, Dijon, France; 13 Service de neurologie, université de Franche-Comté, CHRU de Besançon, 25030 Besançon, France; 14 Department of Neurosciences, Clinical Investigation Center CIC 1436, Parkinson Toulouse Expert Centre, NS-Park/FCRIN Network and NeuroToul COEN Center, Toulouse University Hospital, INSERM, University of Toulouse 3, Toulouse, France

**Keywords:** Parkinson's disease, Prognostic markers

## Abstract

Parkinson’s disease (PD) is affecting about 1.2 million patients in Europe with a prevalence that is expected to have an exponential increment, in the next decades. This epidemiological evolution will be challenged by the low number of neurologists able to deliver expert care for PD. As PD is better recognized, there is an increasing demand from patients for rigorous control of their symptoms and for therapeutic education. In addition, the highly variable nature of symtoms between patients and the fluctuations within the same patient requires innovative tools to help doctors and patients monitor the disease in their usual living environment and adapt treatment in a more relevant way. Nowadays, there are various body-worn sensors (BWS) proposed to monitor parkinsonian clinical features, such as motor fluctuations, dyskinesia, tremor, bradykinesia, freezing of gait (FoG) or gait disturbances. BWS have been used as add-on tool for patients’ management or research purpose. Here, we propose a practical anthology, summarizing the characteristics of the most used BWS for PD patients in Europe, focusing on their role as tools to improve treatment management. Consideration regarding the use of technology to monitor non-motor features is also included. BWS obviously offer new opportunities for improving management strategy in PD but their precise scope of use in daily routine care should be clarified.

## Introduction

Parkinson’s disease (PD) is a neurodegenerative disease affecting almost 1.2 million individuals in Europe and being the second leading cause of motor disability in adults after strokes. In France, there are ~25,000 annual new cases of PD and by 2030, the number of PD patients could increase by 65%^1,2^.

In 2017, a French epidemiological study showed that only 33.5% of PD patients had received a neurology consultation over a 1-year study period^3^, whereas a neurology consultation every 6 months is the recommended standard of care in the French national protocol for diagnosis and management (PNDS). Limited access to neurology consultations could be related to several factors, including the insufficient number of neurologists and unequal repartition over the national territory (French population in 2021: 67,75 millions; French neurologists in 2022: 2980; French neurologists belonging to expert PD centres in 2022 :100). This data reflects a global trend, as in Germany, or in the United States, >40% of PD patients are treated by general practitioners^4^.

As the disease progresses, the response to dopaminergic subsitutive therapy changes. The classical motor manifestations, i.e. bradykinesia, resting tremor, rigidity, and gait disorders will eventually fluctuate. Dyskinesia will occur and the burden of non-motor symptoms (NMS), such as pain, mood disorders, sleep disturbances and dysautonomia, which usually vary during the day, will increase. Thus, PD may be viewed as “fluctuating” disease in nature^5,6^. The management of fluctuations (especially in patients with advanced PD), require frequent treatment adjustments based on expert knowledge of clinical features and available pharmacotherapy. Treatment adjustments can be challenging even for neurologists belonging to tertiary movement disorders centres, as their decision should be based on patients’ subjective feelings and caregivers’ perceptions that are sometimes difficult to collect. Overall, this highlights the need to improve the specialized care offer for PD patients or to find alternative options for treatment management in routine clinical care.

The COVID-19 pandemia gave rise to a considerable expansion of technology-applied to healthcare that has also invaded the field of PD^7,8^. Quantitative parameters assessing motor condition obtained using wearable technology are growingly considered as clinical trials exploratory outcomes^9^. Inertial measurement units (IMUs) are the most used technology for this purpose. IMUs typically consist of a triaxial accelerometer and gyroscope, and sometimes a magnetometer. For PD remote in-clinic or home-based monitoring, IMUs have been embedded in devices worn by the patient (i.e., wearable sensors and systems). The growing number of publications and available body wearable sensors (BWS) for PD, have led the International Movement Disorder Society (MDS) to create a dedicated Task Force, aiming at maximizing the diagnostic and therapeutic potential of technology in the care of patients with movement disorders^10^. In January 2023, the UK National Institute for Health and Care Excellence (NICE) has published their national recommendations regarding the use of devices for monitoring of PD^11^. Although some results are promising, the use of BWS in daily clinical practice has been quite limited and “practical recommendations” are still lacking to ensure the best outcomes for PD patients, their caregivers and clinicians. Additionally, BWS outcomes seem to be influenced by psychological, physiological, cognitive, environmental, and technical factors, with possible inconsistency in mobility parameters when comparing laboratory vs. in-home unsupervised recordings^8^.

This practical anthology aims to summarize the available and most used BWS for PD patients in Europe, monitoring PD manifestations and used for treatment adjustments.We specifically focused on technology for PD treatment management and we do not touch other aspects, even if valuable, as PD early and prodromal detection or the use of BWS as biomarkers in clinical trials. For each BWS strengths and weaknesses, regarding their performances, validation process, ease of use and reports’ clarity have been summarized based on a not systematic literature review and authors’ expert opinion.

The authors of this article are part of the “Wearable sensors workgroup” of the NS-Park French Network.

## Historical perspective: laboratory-based motion capture systems

Some BWS were developed and valitated based on comparisons with laboratory based motion capture systems. Among these systems, the most widely used is Vicon^TM^ (particularly for BWS recording gait parameters). Before entering into the details of BWS, we propose a brief paragraph on the Vicom^TM^ system, to provide an historical perspective on laboratory-based motion capture system applied to PD, as it was one of the first in-hospital product to objectively evaluate PD motor manifestations. The Vicon^TM^ Motion Systems Limited is an optoelectronic motion capture system, which is a set of class I Medical devices with a CE-mark. Multiple spherical retro-reflective motion sensors, typically from 2.5 to 14 mm in diameter, positioned on the face, chest, back, upper and lower limbs of the moving subject are tracked by infra-red cameras positioned on the walls of a dedicated room, in order to generate kinematics parameters. A reconstruction of the position of the different sensors in time and space allows a 3D analysis of the movement. The Vicon^TM^ Motions Systems Limited has the potential to objectively measure the evolution of movements in subjects.

### Validation studies

To study PD gait, the typical “plug-in-gait full body” set is placed in the front and the back of the body^12^, but additional markers can be placed as needed, on the fingers and face for instance, according to the movement studied^13^. The medical device requires a calibration to measure marker position, typically within 2 mm accuracy at a frequency of 100 Hz (lower rates of 50 Hz have been previously used)^12^. The Vicon^TM^ system was usually used as a source of read-outs for basic and clinical studies of gait and movement in PD. In addition, it serves as the gold standard reference comparison for new devices or wearables, in particular in gait analysis or PD patient evaluation^14–16^. Previous studies have shown that the positioning error would be above 1 mm. It corresponds to the error magnitude usually considered as a standard for this type of systems. But others suggest that, if calibrated properly, and given a careful choice of marker sizes, Vicon^TM^ sampling rate, camera sensor resolution and closer camera distance to the tracked objects, the error is <1 mm, in both static and dynamic measurement experiments^17–19^.

### Strengths and weaknesses

This system does not allow remote at-home monitoring of patients in an ecological environement, and requires the patient to come to the hospital. It is considered one of the most accurate motion capture system but it requires the patient to perform specific tasks during a limited time, repeating each measured task several times (usually twice or 3 times) to ensure a proper analysis of the movement. Yet, it is considered the gold standard tool and is widely used as a reference to calibrate and validate other devices’ accuracy, that will be used as wearables, some of them described in the next sections of this review.

## Wearable sensors selection

Technology products have different degree/phase of development reflecting the maturity or “readiness level”, i.e. how close a system is to being validated for use in routine care. The Technology Readiness Level (TRL) scale developed by NASA in the 1970s is a commonly used scale to define the degree of validation of a technology product. For the purpose of this article we have included wearables sensors that hold a TRL of 8–9, meaning that they have sufficiently demonstrated to work in clinical practice or in-home environment^20^ and that are available in Europe and CE mark for clinical use in PD. Thus, the following BWS have been reviewed, regarding product description, validation process, clinical applications and regulatory status: PDMonitor®, Personal KinetiGraph® (PKG®), STAT-ON, Kinesia 360™/One™ and FeetMe®. Additionally, we also describe the “MobilityLab” system, having a TRL of 8–9, even if not used in Europe, as largely validated and FDA-approved for PD axial features monitoring. For the sake of clarity, based on different gold-standard for validation, we considered two distinct groups of BWS: (a) wearable sensors validated vs. patients and clinicians-based scales (PDMonitor, PKG, Sense4Care and Kinesia 360/U); (b) wearable sensors for gait analysis validated vs. laboratory based motion capture systems (FeetME and Mobility Lab).

## Wearables sensors: description, validation process and clinical applications

### PD monitor

**Fig. 1:**
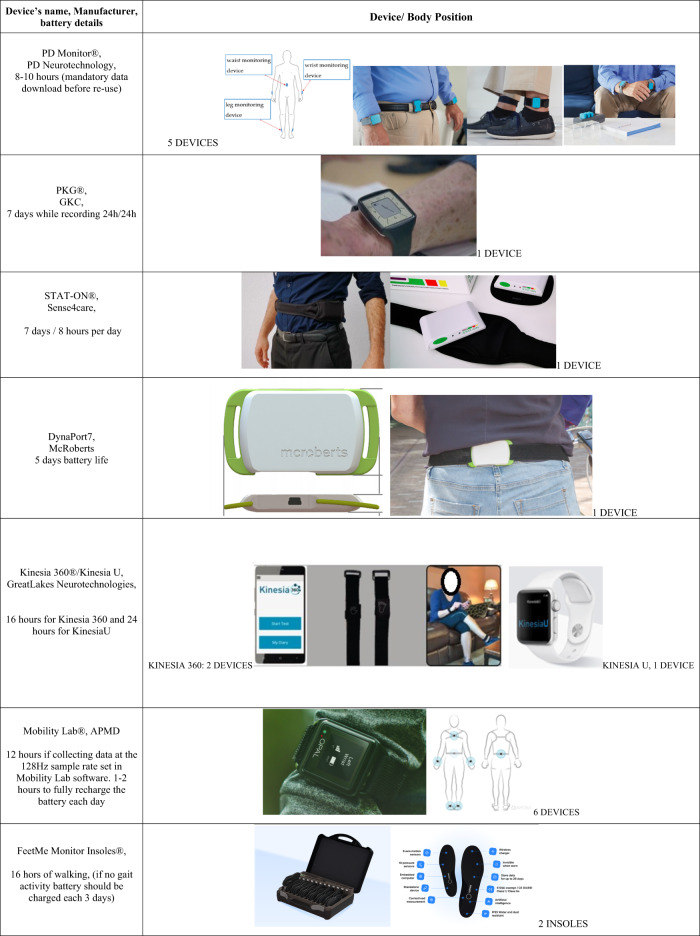
Body worn sensors for Parkinson’s disease management. Authors have obtained consent to publication of the image from PD Neurotechnology Ltd, Global Kinetics, Sense4Care, McRoberts, GreatLakes Neurotechnologies, APDM Wearable Technologies and FeetMe.

PDMonitor®, consists of five identical IMU-based sensing devices, placed on both wrists, shins and waist, designed and developed to monitor patients with PD. The components of the PDMonitor® device are: (i) a SmartBox: used to collect, process and upload data to the Cloud; (ii) 5 sensing monitoring device each with internal data storage for the period they are worn (for a minimum period of 2 h up to 7 days) and transfer the data collected when they are docked into the SmartBox; (iii) accessories used to attach the devices to the patient (Fig. 1).

When the data are docked, they can be transferred as a pdf report to the clinicians (Fig. 1). The following clinical outcomes are present in the report, each measured every half-an-hour; (i) Off-score, expressed as a score ranging from 0 to 1.0; (ii) dyskinesia, expressed as a global score or single score for each arm and leg, each scoring from 0 to 4; (iii) arms/legs bradykinesia and global body bradykinesia, score ranging from 0 to 4; (iv) arms/legs tremor and global body tremor, score ranging from 0 to 4; (v) an estimation of the UPDRS part III score, ranging from 0 to 108; (vi) gait impairment, score ranging from 0 to 4; (vii) FoG index, score ranging from 0 to 1.0; (viii) postural instability, score ranging from 0 to 1.0; (ix) number of steps made during the recording and activity/lack of movement index, as well as sitting time and lying time, ranging from 0 to 1.0; (x) gait analysis including cadence (step/min), gait speed (m/sec), stride length (cm).

### Validation studies

The PDNST001 study assessed the PDMonitor® system among 65 PD patients over an in-hospital recording of 6 h (Phase I) and over a recording of at least 1–3 days at home, during at least 7 h/day (Phase II)^21^. During the Phase I patients have been assessed combining the PDMonitor® with a video-recording of the UPDRS and the Abnormal Involuntary Movement Scale (AIMS), for a third-party physician. Patients were preferably in Medication-Off (Med-Off) condition and a diary was kept by their caregivers or nurses to describe their motor state while performing different motor tasks. During Phase II study, patients were asked to keep a diary. A Comfort Rating Scale was used at the end of Phase II to measure the wearability of the device. As a result, for wearability: (i) patients needed about 5 min to wear the device but were faster with assistance; (ii) the waist device was the most inconvenient. Regarding clinical features detection, a good accuracy was found for: (i) bradykinesia index and UPDRS arm bradykinesia subscore (accuracy: 86%); (ii) dyskinesia index vs. AIMS and gait index vs. UPDRS gait score (accuracy: 99% and 99%, respectively); (iii) tremor index vs. UPDRS item 20 (accuracy: 99%); (iv) FoG presence/absence vs. clinician/patients’ diary (96%). The probability of the patient being OFF, is calculated by the PDMonitor® based on a combination of different index including gait, tremor, FoG and bradykinesia and it has been compared to patients’ diaries finding a high correlation (*r*^2^ = 0.75). Similarly, the dyskinesia index showed a moderate correlation (*r*^2^ = 0.63) with the AIMS.

Another case-control study has evaluated PDMonitor® performances among 61 PD patients and 27 age-matched controls in both clinic and home environment. Authors report on an average accuracy of 99.1% of the sensor in detecting body and arms/legs dyskinesia, but the publication does not clearly explain the gold standard vs. with the sensors have been compared^22^.

More recently, a German pilot study on 12 PD patients with a mean age of 65 years, assessed the feasibility of PDMonitor use over 12 weeks, finding good values (from “Okay” to ‘very good” in a satisfactory Likert scale), but with technical support needed for the whole study period^23^.

Finally, one report on two PD patients described how PDMonitor® could help in the treatment adaptation (levodopa increment) during restrictions in the access to healthcare units, due to the COVID-19 pandemic to better identify Off-periods^24^.

### Strengths and weaknesses

Overall, PDMonitor® has the advantages to propose a home-based continuous monitoring over 7 days, capturing different parkinsonian manifestations (ranging from Off-score, dyskinesia, global motor impairment and gait/axial parameters), offering a clinical report that is sent to physicians but that is quite long (1 first summary page with hour/hour averages for ON/OFF dyskinesia and UPDRS scores and 30 pages for the whole report). Overall accuracy vs. clinical-based score seems to be from moderate to high, but it has been evaluated only in 2 studies included with small groups of patients (61–65 patients) and should be replicated in larger, multi-centre cohorts. Additionally, patients seem to require continuous technical support and PDMonitor® use implies the use of 5 sensors at the same time.

### Personal Kinetigraph

The PKG® technology was developed by neurologists in Australia, in order to quantify kinematics of motor manifestations in the real-life conditions for parkinsonian patients. It also includes a reminder for taking anti-parkinsonian medications, i.e. a medication administration marker when these are taken, a sensor to detect the removal of the watch and it can monitor activity associated with movements during sleep.

The PKG system consists in a PKG watch and PKG report (Fig. 1).

The PKG watch is a wrist-worn medical device, worn on the side of the body most severely affected by PD. It weighs 35 g. It contains a rechargeable battery and a 3-axis accelerometer, sampling rate of 50 samples per second and data storage on flash memory.

It is carried by the patient over 6–10 days. During the wear period, the PKG watch automatically collects data on the type of movement experienced by the patient and reminds the patient to register when they have taken dopaminergic medication as prescribed by their physician. A mathematical algorithm translates the raw movement data collected into bradykinesia and dyskinesia scores and provides a graphical PKG report (Fig. 1)^25^.

The PKG report gives a measure of severity and proportion of time spent at different levels of dyskinesia and bradykinesia in relation of timing of levodopa medication. The main plot of the PKG report shows the median, 25% and 75% percentile of the bradykinesia score (BKS) and dyskinesia score (DKS) of the patient over all days of recording compared with the BKS and DKS of a control group (non-PD patients).

Fluctuations and dyskinesia score (FDS) is a summary score combining variation in bradykinesia and dyskinesia scores and is representative of motor complications.

Other and separate scores are the Percent Time in Tremor (PTT) which is a summary score for the ambulatory assessment of tremor and the Percent Time Immobile (PTI) which represents the percentage of time the patient was totally immobile, helping to describe sustained immobility, daytime sleep and somnolence.

### Validation studies

Several studies have evaluated the clinical performance of PKG vs. patients’ diaries or physicians-based assessment.

For tremor detection the PTT summary score has been evaluated in two consequtive cohort of 85 and 87 PD patients showing similar results: a PTT of ≥0.8% had a 92.5% and sensitivity and 92.9% selectivity, in identifying resting or postural tremor that involved the wrist whose frequency was >3 Hz^26^.

FDS has been validated in a population of 527 PD patients and then further tested in a second group of fluctuators (*n* = 36) and non fluctuators (*n* = 16), showing to differentiate the two groups with a sensitivity of 97.1% versus the gold-standard clinical evalaution (AIMS score for dyskinesia and UPDRS-III for bradykinesia)^27,28^.

The median PKG Bradykinesia was shown to closely correlate with the UPDRS III motor score (minus tremor item) in 25 PD patients with bilateral well established disease (*p* < 0.0005; *r*^2^ = 0.64) and the PKG Dyskinesia closely correlated with the modified AIMS in 34 PD patients (*p* < 0.0001: r^2^ = 0.80)^25^. In an observational study, Ossig, et al. compared the PKG scores to 24 patient home diaries. They reported that global distribution of total hours/day in all motor states measured by PKG reflected those assessed by PD home diaries. However, hour to hour agreement was weak (Cohen’s κ: 0.304), but with a moderate correlation between objective measure of PKG and diary data for Off and On state without dyskinesia, and a strong correlation for the dyskinetic periods (Cohen’s κ: 0.404; 0.562 and 0.658, respectively)^29^. In the qualitative evaluation of Santiago et al., movement disorder specialists in a PD clinic, reported that PKG provided additional information beyond that obtained during clinical consultation alone, in 41% of visits, and resulted in adjusting treatment nearly a third of the time overall^30^. The PKG most yielded new and accurate information about daily off-time (50% of cases)^30^. A blinded controlled trial conducted in 2020 in 154 PD patients, compared physician management of PD using information provided by objective ambulatory measurements and conventional assessment, with management using conventional assessment alone. This study shows that therapeutic decisions supported by objective measurement (PKG+ arm) resulted in reduced bradykinesia, motor complications and improved global motor disability as measured by the MDS-UPDRS Total score, part III and IV, with no changes in PDQ39^31^. In contrast, the changes in MDS-UPDRS Total, MDS-UPDRS part III, and PDQ39 in the PKG—arm were not statistically significant.

Once evaluating satisfaction, in a study including 65 PD patients (mean disease duration of 10 years), patientes stated in 82% of responses that they agreed or strongly agreed in PKG training, usability, performance, and satisfaction^32^.

Finally, regarding NMS, the PTI has been suggested as a surrogate marker of daytime sleepiness, has shown a good correlation with ambulatory daytime polysomnography (85.2% concordance) and high Epworth Sleepiness Scores (*p* = 0.01)^33^ (*see paragraph on NMS*).

### Strengths and weaknesses

PKG has the strength to be only one device easy to wear. It is able to detect motor fluctuations/bradykinesia, dyskinesia and, offering probably an measure of day-time sleepiness. The graphical report is easy to read offering a summary of the whole period in one unique graphic indicating the changes in bradykinesia, tremor and dyskinesia, immobility/inactivity (including night period) as well as medication intakes. PKG has been included in 26 studies with large sample size (up to 3288 patients for the largest study) of PD patients and compared vs. patients’ diaries or physicians-based assessment^26,29–32,34–55^. It has been compared as add-on tool compared to conventional management in a randomized trial^31^ with some positive results and as add-on tool to select PD patients for device-aided treatment^50,51^. Indeed in 2018 an expert review suggested that PKG increases clinical decision-making, and that the objective measures of the PKG could confirm the presence or the absence of motor manifestations as reported by the patient^27^.

PKG has some limitations: the device measures only some of the motor PD manifestations, only on one extremity and does not directly evaluate gait, FoG and falls.

### Stat-On^TM^

This is a wearable sensor analysing inertial signals, with a set of validated machine learning algorithms running in real time. This sensor measures 90 mm × 62.5 mm × 21.2 mm and weighs 86 grams (Fig. 1). Internally, the system is composed of two ultralow triaxial nano-accelerometers, two microcontrollers, and a Bluetooth Low Energy system. The sensor has a battery life of 7 days for a continuous 8 h/day. The compromise in this tool was to rely on a single sensor to ensure usability and to locate the system over the waist given that it is very close to the mass centre of the body and many movements, at least partially, are reflected there (Fig. 1). Information is provided on several parameters of gait, such as step length, FoG and falls, as well as upper and lower limbs movements, trunk or neck dyskinesia, while bradykinesia is mainly estimated from gait and tremor is not recorded by the system^56^.

### Validation studies

STAT-ON^TM^ has the aim to evaluate ON/OFF fluctuations and gait changes over seven daysand it has been compared with^57^ Hauser diaries. Four clinical studies have compared STAT-ON^TM^ with patient self-report of ON/OFF status^58–60^; in one study an observer stayed with the patient, during several hours^60^. A total of 74 patients were included, one study being multicentre over four countries^58^. Considering technical aspect of drop-off related to different reasons, a total 53 fluctuating patients were analysed with recordings of various duration from several hours to 3 consecutive days. Accuracy was compared to: (i) patients diaries with 30 min intervals; (ii) report from phone interview, every two hours; (iii) report of observers either over several hours or once a day. Altogether, the detection of ON and OFF periods in the three studies had a sensitivity ranging from 92% to 97% and a 88–94% specificity. Nevertheless in one recent paper including 39 PD patients, Cohen’s κ agreement analysis between the UPDRS-IV/clinical interview and STAT-ON was low for motor fluctuation (0.089) and fair for dyskinesia (0.318) and FoG (0.481) ref. The same study evaluated patients satisfactionIn one paper, these scores were obtained by restricting analysis to data obtained with at least 10 consecutive strides^58–60^.

The validation of bradykinesia measurement was based on two studies with very similar approaches^61,62^. The main difference is that the first pilot study included 12 PD patients and the gold standard of bradykinesia measurement were home-video recordings of movements over 30 min ON and 30 min OFF, whereas the second was a multicentre study including 75 patients with only UPDRS measurements of motor status. In both studies, patients performed a mixed of freely and requested task, in which gait took a large place. In the pilot study, the average specificity and sensitivity in detecting bradykinetic gait was 89.07% and 92.52%, respectively, while the average accuracy was 91.81%^61^. In the larger study, the correlation between the total UPDRS-III score and bradykinesia score provided by the STAT-ON^TM^ was significant *(ρ* = −0.56, *p* < 0.001) as well as the one with with the gait item of UPDRS (*ρ* = −0.729, *p* < 0.001), while a less significant correlation was found with hand rest tremor (*ρ* = −0.182, *p* = 0.026).

The assessment of peak-dose dyskinesia was also performed in a multicentre study in which 35 of 92 patients had such dyskinesia^59^. STAT-ON^TM^ system detects any dyskinesia of the trunk with a sensitivity over 93% and 95–98% specificity. Conversely, when dyskinesia only involve the limbs without inducing trunk movements, sensitivity drops to 39%, although specificity remains high (95%). Likewise, in a subset of 13 patients video-recorded whose dyskinesia were evaluated using the Unified Dyskinesia Rating Scale (UDysRS), the correlation was significant between UDysRS and STAT-ON^TM^ measures for the trunk and legs (*ρ* ranging from 0.64 to 0.77, *p* < 0.025) but not for arms or neck dyskinesia (*ρ* ranging from 0.25 to 0.53, *p* > 0.14)^63^.

Step lengths has been assessed using STAT-ON^TM^ system in 28 patients in different conditions, i.e., ON or OFF and compared with video recordings of the patients. The tool achieved a 96.8% accuracy in step detection observed on video recordings^64^. However, this level of accuracy is obtained with individual correction factors whereas a generic correction factor has been proposed to avoid an individual calibration process.

FoG was assessed in a subset of 21 patients included in one of the previous study^65^ whose FoG questionnaire score^66^ was 6 or higher [7–23]. Patients were examined during ON and OFF medication conditions, at home. A total of 93.03 min of FoG episodes were registered (1321 episodes), among which 74.12 min (79.67%) were registered when patients were in OFF state, 12.35 min (13.28%) were registered when patients were in ON state and 6.56 min (7.05%) were registered in an intermediate state. The ability of the system to identify FoG has a sensitivity of 79% and a specificity of 75% compared to physician-based observation at home^65^. Falls are also detected with a reported 95% sensitivity and 99% specificity compared to clinical obervation, but full data on this study are not yet published (reported in^56^).

Finally, the STAT-ON^TM^ system is intended to identify postural changes of PD patients, with a good accuracy, both in ON (98.5%) and OFF (97.6%) state, in particular when changes between sit and standing positions occur, although dyskinesia may create some bias in the analysis^60,67^.

### Strengths and weaknesses

Among the strengths of STAT-ON^TM^, we can mention the use of a single sensor, and an acceptable usability for patients. In addition, the “event” button can provide personalized information such as drug intake, which is useful for further analysis of patients’ status. The feedback provided by the software to the physicians is easy to read and help to better analyse daily-living condition of the patient (Fig. 1). STAT-ON^TM^ performances in regard to PD clinical features have been evaluated in 9 studies, one beeing multicentre.

However, some limitations desserved to be mentioned. Considering the position of the sensor, postural information and gait are captured but bradykinesia is best recorded during walking, ideally with at least 10 consecutive strides. In addition, dyskinesia of the trunk and legs are accurately measured which is not the case for peripheral limb choreic dyskinesia, especially if they are mild and isolated. Eventually, methodological issues remain. The numbers of patients, who were involved in the various processes of validation are low with no comparison to healthy controls in most reports. Still, when conflicting interpretation between patient and observer about a patient condition occur, the corresponding data are discarded from analysis, which may *de facto* limit the performance of the system to detect intermediate clinical status. Finally,STAT-ON report does not offer a graphic summary of parameters’ averages of the whole recorded period but averages of daily total OFF time and average of daily FoG episodes (one mean for each day) and a detail of each single day including all features (FoG, dyskinesia, OFF, falls and medication) in one line (each day = one graph = one line, which means 7 lines for 7 days). A user with STAT-ON can download a report which might be basic (5 pages) or extended (length depending on the number of monitoring days, it can be >40 pages).

### Kinesia^TM^ technology

Kinesia ONE^TM^ (Cleveland medical Devices Inc., Cleveland, Ohio, USA) includes a wearable sensor, an iPad mini preloaded with the kinesia ONE application and a charge pad. The motion sensor contains three orthogonal accelerometers and three orthogonal gyroscopes to measure linear accelerations and angular velocities respectively. The sensor is worn on the finger index or on the heel during specific tasks used to assess motor manifestations (tremor, bradykinesia, dyskinesia, gait, freezing of gait). A mobile application guides patients through doning the motion sensor and provides instructions for completing the assessment tasks based on common rating scales (e.g., UPDRS for Parkinson’s disease, TETRAS for essential tremor). Once an assessment has been completed, motion data is transmitted to a secured cloud database (the Kinesia Web Portal) and algorithms are used to calculate severity scores on a 0 (no signs) – 4 (severe signs) rating scale shown to be correlated with clinician ratings. Clinicians access the data and reports by logging into the Kinesia Web portal.

Kinesia UTM includes a mobile application and a smartwatch worn on the patients’ wrist to quantify tremor, bradykinesia and dyskinesia during specific tasks or throughout the day during normal activities.

Kinesia 360™ includes a mobile application and two wireless motion sensors worn on the patient’s wrist and ankle (Fig. 1) to quantify tremor, bradykinesia, dyskinesia, body position and steps throughout the day during normal activities. For Kinesia UTM and Kinesia 360™, electronic diaries are included in the application for patient rating their symptoms and log when medications are taken. Data from the motion sensors are uploaded to a secured cloud database and algorithms are used to detect clinical features and calculate severity scores every 2 min on a scale. Motor symptom reports are generated from the cloud server for clinician review. Sensors record data all day and recharge overnight for extended home use.

### Validation studies

The efficacy of Kinesia ONE^TM^ for recording kinematics of tremor in PD was evaluated in a study including 60 PD subjects^68^. In this study, PD subjects performed the tremor subset of the UPDRS motor section while wearing the device. Each subject was seated in front of a computer screen and instructed to perform three tasks evaluating rest, postural and kinematic tremor. Data were wirelessly transmitted from the patient-worn unit to the computer. Each trial was videotaped and videos presented to two movement disorder neurologists for scoring according to the UPDRS motor section task 20 for each tremor task. After clinical data collection, clinical acceptability was evaluated by patient questionnaires. The Kinesia system captured biokinetic data related to tremor severity. Correlations using the device were high for rest tremor (*r*^2^ = 0.89), postural tremor (*r*^2^ = 0.90), and moderate for kinetic tremor (*r*^2^ = 0.69). The wireless link provided real-time synchronization between video-instructed tasks and data analysis. The quantitative features recorded were used to develop a mathematical model that predicted tremor severity scores. Forty PD patients completed the questionnaire of clinical acceptability. All of them found the device comfortable, lightweight and unobtrusive.

The Kinesia system also successfully captured biokinetic data related to bradykinesia severity, according to the Modified Bradykinesia Rating scale (MBRS), speed amplitude, and rhythm scores^69^ among 50 PD patients, with a moderate correlation (r^2^: 0.63–0.67) among kinematic outcomes and MBRS sub scores.

The feasibility and compliance of patients to use this device at home was evaluated in a pilot study in 10 parkinsonian patients at early stage of the disease^70^. Subjects used the system at home to perform 6 video-guided motor tasks per day for 3–6 consecutive days, while motion sensor data were captured to evaluate tremor and bradykinesia in response to PD medication. The authors demonstrated that PD patients were able to perform correctly motor tasks based on the UPDRS unsupervised at home to evaluate tremor and bradykinesia of the more affected hand. Nevertheless, the device failed to detect properly tremor and bradykinesia when the two features overlapped.

#### Kinesia UTM

It has been tested in a small study including 14 PD patients, whose motor features (tremor, slowness and dyskinesia) were assessed for 1 week prior to instituting a doctor recommended change in therapy and for 4 weeks after the change. Thereafter, patients were seen by the clinicians who made therapy recommendations based on the reports and his clinical judgement. The objective changes highlighted by Kinesia UTM were compared to qualitative clinicians judgement, finding an improvement in at least one symptom measured by the BWS in the eight participants who were deemed by the clinician to have improved, and an aggravation in two of the five patients considered as “worsened” by the clinician^71^.

#### Kinesia 360^TM^

The validity of this device has mainly been studied to assess tremor, bradykinesia and dyskinesia. It was evaluated in a study including 13 PD patients, with a history of motor fluctuations and levodopa-induced dyskinesia^72^. All the subjects were instrumented with the device and recorded by video. Kinematic data were recorded during a two-h series of activities of daily-living in a simulated home environment through transition from OFF to ON medication. Algorithms were applied to the kinematic data to score tremor, bradykinesia and dyskinesia. A blinded clinician rated the severity of the clinical features using the UPDRS III observed on video. Algorithms scores for tremor, bradykinesia and dyskinesia agreed with clinician ratings of video recordings with a high correlation coefficient (ROC area > 0.80). In 2019, a 12-week pilot study investigated whether using Kinesia at home could improve motor symptom management in PD patients starting transdermal dopamine agonist^73^. 39 PD patients were included and randomized 1:1 to control group (CG) or experimental group (EG). Motor manifestations were assessed at baseline and week 12. At week 12, mean improvements in UPDRS II and UPDRS III were clinically greater in the EG, and mean number of dosage changes during the study was higher in the EG versus CG. Tolerability and retention rates were similar. The authors concluded that continuous motor manifestations monitoring using the device may enhance clinical decision-making and improve motor outcomes in PD patients starting this therapy.

### Strengths and weaknesses

Overall, Kinesia^TM^ Technology has been adopted in 19 studies including PD patients^68–86^. Kinesia ONE™ quantifies tremor, bradykinesia and dyskinesia in PD patients under standardized motor tasks with a good sensitivity and sensibility, as evaluated in 9 studies (sample size: 2–85). The device is light, not-binding and well-accepted. Nevertheless, it does not make it possible to evaluate the motor manifestations of the patients during routine activities. It cannot therefore constitute a tool for evaluating nor improving the therapeutic management of PD patients in real-life conditions.

Kinesia UTM and 360™ has the advantage of being able to monitor the patient’s motor status continuously and in real-life conditions. It is particular sensitive and specific for evaluating tremor and dyskinesia. Its specificity for detecting bradykinesia is lower and some items of the UPDRS III are note captured, such as rigidity or posture. Kinesia 360™ has been evaluated in 10 studies with smal sample size (range: 13–60 patients). The device is easy to install, well-accepted and the graphic reading of the results for the practitioner seems easy to read, representing bradykinesia, dyskinesia and tremor fluctuations on the same graphic with different colours, with one graphic for each day.

### DynaPort

The DynaPort7 (Mc Roberts) is a small, light-weight sensor on a belt on the lower back and it is the new version of the DynaPort Hybrid system. It combines acceleration sensors and gyroscopes with six channels that assess the individual’s movement at 100 Hz each. It uses Bluetooth protocol, to communicate with a host personal computer. Quantity measures include: the total number of walking bouts, the total number of steps, median walking bout duration, median number of steps, variability of the gait pattern, gait rhythmicity, gait smoothness, the phase coordination index (measure of the consistency and accuracy of the left-right bilateral coordination during walking) and median cadence per bout and the percent of time spent walking which reflects the total walking in relation to the overall walking and non-walking activity. Mobility classification is done from second to second, and report can present data with mean per day or per week.

### Validation studies

Validation and acepptability studies in PD have been made on the first hardware unit which was the DynaPort Hybrid system, not on the lastet DynaPort7 version.

The algorithm was developed in a cohort of elderly 350 elderly patients, but neither dementia, nor PD and showed to well identify misstep events with a good hit ratio and specificity (odds ratio was 1.84, 95% confidence intervals: 1.15–2.93)^87^. When tested in PD cohorts the algorithm showed that PD fallers have a different gait quality if compared to non-fallers (sample size: 107 patients)^88^, with higher percentage of missteps if compared to non-fallers, in another cohort of 40 PD patients (*p* = 0.01)^89^. In an in-hospital assessment of 176 PD patients, seven of the twelve mobility measures were significantly associated with the MDS-UPDRS part III after adjustment for age, sex, and disease duration (0.0001 < *p* 0.005)^90^. Finally, DinaPort has been also used to characterise in-home physical behaviour comparing 17 PD not cognitively impaired (PD-NC) with 22 PD with mild cognitive impairment (MCI) with 9 PD with dementia (PDD). PDD patients showed fewer but longer sedentary and sitting bouts than PD-MCI (*p* = 0.01/0.03) and PD-NC (*p* = 0.01/0.04)^91^.

### Strengths and weaknesses

DynaPort is a single small device, easy to wear, able to give information about gait parameters and risk of falls. McRobert technology also includes two other products, i.e. MoveMonitor and MoveTest, able to monitor physical activity over 2 weeks and to do short physical test performance, respectively. A study assessing wearable acceptability showed that all included 18 PD patients considered DynaPort as both comfortable and acceptable^92^. However, DynaPort does not provide information on other parkinsonian manifestations, including tremor, dyskinesia and motor fluctuations and its’ performances have been evaluated only in three studies specifically including PD patients.

### Feet me monitor

Feet Me® Monitor is a three part solution (insoles, connector and App) used for continous remote and real time monitoring. It allows healthcare professionals to collect mobility data from remote patient monitoring to assess the level of disability and risk for falling, but also patients progress and response to treatment. Connected insoles are instruments used to perform proprioceptive training using biofeedback or to collect balance and gait parameters for monitoring gait and posture.

FeetMe® Monitor connected insole is a wearable medical device that measure spatio-temporal gait parameters and plantar pressure to support the mobility assessment of individuals. The hardware product consists of two regular shape insoles and a double-head wireless charger with wall adaptor to charge both insoles simultaneously (Fig. 1). The insoles are available in 12 sizes ranging from 35 to 46 (EU standard) or from 4.5 to 13 (US standard for men) or 5.5 to 14 (US standard for women).

An insole includes one IMU with a 3-D accelerometer and a 3-D gyroscope to capture movement in 6 directions and eighteen capacitive cell pressure sensors, around 15 mm² cell area each. In the insoles key parameters of the gait cycle (namely heel strike (HS) and toe off (TO)) events are detected by the pressure sensors and the related temporal parameters are calculated from HS and TO event timings. HS, TO and stride length are calculated in each insole and are defined as insole-based parameters. For each insole, these parameters are sent to a mobile application via Bluetooth Low Energy. The velocity, stride length, stride duration, stance duration, swing duration, step duration, single support duration and double support durations, cadence, number of steps, distance, duration of walking test are defined as mobile-based parameters and are calculated by a dedicated mobile application. The mobile application allows the real-time recording and/or visualization of the gait parameters on the Feet-Me mobility dash-bord, including selected gait parameters reports and actimetry variations The report is customized for each study, and contains both average values over several days of analysis and instantaneous values on the graphs. The usual gait analysis parameters are included: pace, rhythm asymmetry, variability, posture control and walking dynamics. Tests data (e.g. 6 min) are reported, and performance can be compared with data from healthy individuals in the same age group.

### Validation studies

Previous validation studies, demonstrated an acceptable validity and reliability of the collected gait parameters in middle-aged^93^ and community-dwelling older adults^94^ as well as in post stroke and multiple sclerosis patients^95,96^.

In addition, twenty-five PD patients completed two walking assessment sessions. In each session, participants walked on an electronic pressure-sensitive walkway (GaitRite®, CIR System Inc., Franklin, NJ, USA) without other additional instructions (i.e., single-task condition) and while performing a concurrent cognitive task (i.e., dual-task condition). Spatiotemporal gait parameters were measured simultaneously using the pressure-sensing insoles and the electronic walkway. The validity results showed very high correlations and good agreement between the 2 systems, i.e. FeetMe vs. GaitRite (correlation coefficients ranged from 0.73 to 1.00 in both single and dual-task conditions)^97^. Concerning test–retest reliability, excellent correlation (intraclass correlation coefficient laying between 0.8 and 0.95) was demonstrated between the 2 systems.

### Strengths and weaknesses

Feet Me Monitor is promising tools to evaluate gait parameters and help PD patients in the everyday management. FeetMe® insoles (FeetMe, Paris, France) integrate IMU and pressure sensors inside the soles and do not present wires or other external modules, visible over the clothes, as occurs for other instrumented insole systems, such as Medilogic, LoadSole®, Tekscan, and Pedar system^98,99^.

Furthermore, they do not require a connection box connected to a computer to transmit data and they have a real-time feedback of the gait parameters on the smartphone application, contrary to what occurs for PodoSmart and Moticon OpenGo insoles^100,101^.

FeetMe® insoles compute spatio-temporal gait parameters through embedded algorithms and collect them through a smartphone application^93^. All these characteristics lead to better ergonomics, portability, and ease of use, that could enhance the end-users’ compliance and acceptance to wear over long-term monitoring^102^. Hence, the FeetMe® technology allows the use in both clinical and free-living settings.

However, clinical data on PD are still limited, i.e. only one study performed on PD patients, and additional studies and protocols are required to specify the way to monitor patients at home^103^. Indeed, Feet Me cannot offer data on tremor, but a study aiming to detect On-Off fluctuations based on FeetMe gait analysis is actually on-going. Finally, size of the insoles is specific to each person, which makes it not interchangeable with other patients unlike above mentioned BWS (that are intercheangable).

### Mobility lab

Mobility Lab™ by Ambulatory Parkinson’s Disease Monitoring—APDM (http://www.apdm.com/ mobility/) specifically monitors the quality of gait and balance by means of synchronized, wearable inertial sensors, applied in the upper and lower part of the body (Fig. 1). Mobility Lab™ includes: (i) a set 1–6 wireless, body-worn Opal™ inertial sensors (other Opal products/systems that can use fewer or more Opal sensors to collect raw IMU data or kinematic data); (ii) an access point for wireless data transmission and synchronization of the independent sensors; (iii) a software to guide the user and patients through the testing protocols; (iv) automated analysis and reporting of the recorded data, that are immediately available for the users (both patients and investigators) after each test, with comparisons done vs. the previous assessment.

Investigators can choose which set of clinical tests to perform, including the Time up and Go (TUG), 2-min walk, 360° Turn Test, Postural Sway testcos and Balance Error Scoring System (BESS) test. Each Opal^TM^ includes three-axis accelerometers and gyroscopes and a magnetometer, with a 12-h battery and possibility for data storage during a month. User can also hold them during several hours, without doing specific test. However, it this case, only raw data in excel form will be available, without comparison with normative performance. Conversely, those sensors are often used in a research setting to perform the above cited test, possibly in a longitudinal way. Patients can use from one to six Opal^TM^ at the same time, depending on the suitable measure and test: (i) for balance and postural transition measures, one posterior sensor on the lower back (at the level of L5); (ii) two sensors on the feet for gait measures; (iii) two sensors on the arms and one on the sternum for arm range of motion and turning during gait (Fig. 1).

Mobility Lab^TM^ can be used in a clinical and research setting.

### Validation studies

Mobility Lab™ measures for gait, postural balance and arm swing have been obtained in healthy subjects of difference ages to determine reference values^104^, in patients with multiple sclerosis^105^ or mild brain injury^106^, ataxia^107^ and in PD patients, in different disease stages and medication conditions^108–111^. There are at least 30 indexed original studies that have used to investigate Mobility Lab™ performances in PD patients^112^. We summarize the results of some of those, including the original princeps paper on objective measures of balance and gait and correlation with disease severity^111^.

Overall, Mobility Lab™ has been showed to be able to: (i) detect subtle parkinsonian gait in early PD patients; (ii) to evaluate parkinsonian gait/posture responsiveness to levodopa; (iii) to evaluate the global impairment of gait and balance, that has been found to have a moderate to high correlation with disease progression. Indeed, trunk acceleration, as measured by postural sway measures, has been revealed in 13 early untreated PD patients, if compared to 12 age-matched controls^113^; their test–retest reliability was also confirmed in another small case-control study, with a moderate intra-class correlation (0.55 to 0.84 in subjects with PD and 0.60 to 0.89 in healthy controls) and moderate correlation with the postural instability and gait disorders score (PIGD) of the UPDRS-III (*r*^2^ ranged from 0.50 to 0.63; 0.01 < *p* < 0.05). Similar findings have been highlighted in a larger study, having included 135 PD patients and 66 age-matched controls, confirming that TUG and instrumented test of postural sway (iSWAI) measures correlated well with disease severity (MDS-UPDRS-III and PIGD scores)^111^. In a population of 104 PD patients belonging to different disease stages, Mobility Lab™ was able to identify difference motor performance comparing the Med-Off status vs. the Med-On status, with an improvement of arm swing followed by pace-related gait metrics (stride velocity, stride length, and lower leg range of motion) after medication intake, while detecting an increment of sway velocity (worsening of balance) in the Med On, possibly related to dyskinesia^114^.

As mentioned, Mobility Lab™ is primarily used for in-hospital assessment, but this single-event mobility measures may not accurately reflect functional mobility during daily life at home. More recently, Opal™ sensors have been used for continuous in-home monitoring, to monitor the quality of turning and the amount of steps/day. Turning quality resulted to be correlated with disease severity (UPDRS-III) and to identifies fallers from non-fallers PD patients in a small study (34 patients)^115,116^. On the same path, Mobility Lab™’s ability to detect FoG episodes has been evaluated in a clinical setting and compared to blinded clinical raters, with a high agreement (*r*^2^: 0.839–0.875) for FoG episodes longer than 2–5 and >5 s, respectively, while a low agreement for shoter FoG episodes (<1 s; *r*^2^: 0.39)^117^. The sensors have shown to be able to differentiate patients with from patients without FoG, over a 7-day in-home recording, based on the percentage of FoG episodes^117^.

Finally, a second version of Mobility Lab™ that principally applies sensors to feet rather than to shanks, has been recently validated among 21 PD patients vs. pressure sensor walkway, showing a high correlation (*r*^2^ > 0.75) for several gait parameters (gait velocity, stride length, stride length, cadence, stride time and stride time)^118^.

### Strengths and weaknesses

Mobility Lab™ is one of the best validated tools in terms of study number for gait and postural in-hospital analysis for PD patients, whose parameters are also able to differentiate between the Med-On and Med-Off conditions.

However, it is mainly used for in-hospital assessment, it does not capture tremor and its feasibility in continuous in-home monitoring still need to be proved in large cohorts. Mobility Lab™ report is also quite complex to read, individually and graphically representing each gait parameter or offering gait data in an excel table, that need interpretation by trained physicians.

## Regulatory situations

See Table 1 and Table 2 for summary on devices target clinical features, validation studies, regulatory situations and intended approved use, which are quite heterogenous comparing the reviewed BWS. Overall, in Europe BWS are essentially adopted in research clinical studies or lend by pharmaceutical or healthcare service providers involved in the routine treatment of advanced PD patients. The only available European guidelines for BWS are NICE reccommendations that recently indicated Kinesia 360, KinesiaUTM, PDMonitor, PKG and STAT-ON as “conditionally recommended options for remote monitoring of PD to inform treatment”, though not expressing any indication on one wearable vs. another neither on frequency of use^11^. All the revised BWS belong to medical Class I (Im = device with low risk with “m” for measure) or medical Class IIa (with low to medium risk, installed on the body during 30 min up to 60 days). Instruction for use can be downloaded or requested at each manufacturer web site.

**Table 1:** Overview of body wearable sensors for PD management.

Device name	Target symptoms Falls detection Medications intakes note	Validation process in PD populations		PD Patients’ interaction (active/passive) & clinical acceptability	Regulatory situation/Intended approved clinical use
		Study numbers, PD population (size, age, HY, disease duration)*, accuracy/correlation	Gold standard used		
PD Monitor®	OFF score Dyskinesia Tremor FoG index, Postural instability, Gait analysis (Cadence, gait speed, stride Length) Fluctuations No falls detection Medication intake note by means of phone app	Two studies (sample size: 61–65 patients) Age range: NA; Disease duration: 8.8 ± 4.9 years High correlations with video-recorded UPDRS and AIMS for bradykinesia and dyskinesia, very high for Off-time (accuracy: 99% resting tremor, 96% FoG)	Patients’ diaries AIMS scale (physicians based)	Patients may interact with mobile app to insert antiparkinsonian medications and hours Acceptability investigated among 12 PD patients (from “Okay” to ‘very good” in a satisfactory Likert scale), but with technical support needed for the whole study period	CE mark class IIa / To monitor, record, treat and store a variety of motor and non-motor symptoms frequently occurring in people with PD through the continuous use of a set of portable monitoring devices.
PKG®	Severity and proportion of time in dyskinesia, bradykinesia, Tremor, percent time Immobility, Medication intake markers No falls detection	26 studies, large sample size (max sample size: 3288 patients) Age range: 30–80 yrs; Disease duration range: de novo (2 months)—23 years; HY: 1–4 (but disease data are not reported in all studies) Moderate correlation with bradykinesia, high correlation for dyskinesia, and tremor One study suggesting the PKG help in therapeutic management	Patients’ diaries Clinical scales	Patients may interact with the wearable by pushing a button when the alarms ring indicating a medication intake. In a study with 65 PD patients 82% stated that they agreed or strongly agreed in PKG training, usability, performance, and satisfaction	CE mark class Iia / To quantify kinematics of movement disorder symptoms in conditions such as PD, including tremor, bradykinesia and dyskinesia. It includes a medication reminder, an event marker and is intended to monitor activity associated with movement during sleep.
STAT-ON®	Dyskinesia (trunk, neck) Bradykinesia Parameters of gait: step length, FoG, Falls detection (by the patient) Medication administration marker	9 studies (12–75) Age range: 59–83; disease duration: 5–18 yrs; HY: 1–4 3 studies with good accuracy for On/Off fluctuations detection if compared to patients diaries, 2 studies (12–75 patients) with moderate/high correlation vs. UPDRS-III and gait, respectively; one study with moderate correlation with trunk/leg dyskinesia; one study (39 patients) with fair agreement for dyskinesia and FoG but low agreement for motor fluctuations if compared to UPDRS IV	Patients’ diaries Clinical scales	Patient may interact, pushing a button for medication intakes and falls detection. Satisfaction among 39 pts: QUEST scoring 4 (“quite satisfied”) and 5 (“very satisfied”) for all patients, except for the item “easy in adjusting” (47%), which had a lower score	CE mark Class Iia / Waist-worn inertial recorder, configured by a doctor and used by the patient for clinical, ambulatory, or home environments, that collects the results of the motor disorders and events of the PD patients in a period of time
Kinesia360®	Tremor (resting, postural, kinetic) Bradykinesia and dyskinesia KinesiaU app can be programmed to remind patients to take their medication	10 studies (13–60 patients) Age range: 46–85 yrs; disease duration: 2–31 yrs; HY: 1–4 (clinical data not reported in all studies) high correlation for resting/postural tremor, dyskinesia and bradykinesia vs. physician-based video recordings	Patients’ diaries Clinical scales	No interaction with patients for KINESIA 360 (KINESIA ONE provides task-based motor assessment and for Kinesia U patients need to indicate in the app that they actually did take their medication) No published data on satisfaction/usability (only internal data)	CE mark, Class I, FDA authorization / To monitor physical motion and muscle activity to quantify kinematics of movement disorder symptoms such as tremor and assess activity in any instance where quantifiable analysis of motion and muscle activity is desired. In Canada, Kinesia systems may only be used for therapeutic (not diagnostic) purposes of non-serious situations or conditions. In the EU, KinesiaU™ motor assessment system may only be used to monitor physical motion and activity that are not physiological processes and may not be used for diagnostic or therapeutic purposes.
DinaPort	total number of walking bouts, the total number of steps, median walking bout duration, median number of steps, variability of the gait pattern, gait rhythmicity, gait smoothness	3 studies (40–176 patients) Age range: 41–81; disease duration: 1–14 yrs; HY: 1–4 Disease severity (MDS-UPDRS) correlates with several gait parameters and misstep with history of falls (*p* = 0.01)	Clinical questionnaires	No interaction with patients for DinaPort7 (interaction for short physical test with DinaMove) Considered both comfortable and fully acceptable in a study including 18 PD patients	CE mark Class II in US and Classv I in EU FDA authorization / To collect data on clinical mobility

Wearable sensors for gait analysis validated versus laboratory-based motion capture systems					
FeetMe Monitor Insoles®	Balance and gait parameters (Cadence, velocity, stride length, stride duration, numbers of steps, postural sway)	one study (25 patients) Age (mean): 69 yrs; disease duration: 4 yrs; HY: 2 high correlation for gait parameters between pressure-sensing insoles and the electronic walkway One study on-going aiming to correlate gait parameters with FeetME vs. On/Off fluctuations	GaitRite®	No interaction with patients for FeeMe, but the real-time recording and/or visualization of the gait parameters by means of phone app individualized homecare rehabilitation by means of FeetMe Rehab No published data on satisfaction/usability (only internal data)	CE-marked class Im / For real-time visualization and/or recording of spatio-temporal gait parameters and plantar pressure to support the mobility assessment of individuals
APDM®	gait, postural transition, balance arm swing	At least 30 studies (max sample size: 198 PD patients) Age range: 52–78 yrs; disease duration: 1–19 yrs; HY: 1–4 Moderate correlation with physicians’-base scales (UPDRS-III, PIGD score) High correlation with FoG episodes longer than 2–5 and >5 s Able to differentiate fallers vs. non fallers	gold-standard laboratory metrics (GaitRite Mat,Optical MoCap, Force Plate)	The user chooses which set of clinical tests to perform, including time-up and go,2-min walk, and postural sway tests and receive the objective measures immediately after each test is performed No published data on satisfaction/usability	FDA authorization Class II / To document physical movement (motion detection, quantification of motion, quantification of movement, and quantification of human movement) associated with applications in research and engineering

Correlation coefficient levels: 0.90–1.00 Very high; 0.70–0.90 High; 0.50–0.70 Moderate; 0.30–0.50 Low ; 0.00–0.30 Negligible. Medical class: Im device with low risk (m for measure); IIa = with low to medium risk, installed on the body during 30 min up to 60 days. *FoG* freezing of gait, *PIGD* postural instability and gait disorders, * if an information on clinical features is not reported in the table, it is not reported in the literature, *QUEST* Quebec User Evaluation of Satisfaction with assistive Technology questionnaire, *yrs* years.

**Table 2:** Potential values, shortcomings and unknown aspects of body worn sensors in the monitoring and management of PD.

Potential value of technologies applied to PD management	Risks, Hot-points, shortcomings and unknown aspects
Objectively, continuous, remote monitoring of PD symptoms over several days	Induce a risky reduction of the frequency of face-to-face appointments (helpful to have a complete clinical picture and avoid patient’s isolation)
Possibly helpful in informing on treatment changes	Remote assessment cannot replace in-person visit, just be an add-on to the actual standard of care
Possibly helpful in identifying patients who need treatment changes	Need for continuous technical support and patients being familiar with technology
Helpful in better identifying symptoms for PD patients with/without caregivers or with caregivers not able to help in symptoms recalling	Hardly applicable to demented patients or patients with severe mobility impairment (HY 5)
+/− Reduction of the need to travel to hospital and associated costs	No data compared different wearable sensors: not known which is the one with highest accuracy (highest performance, highest wearability) and the most suitable for a specific patient/symptom
+/− Reduce the length and number of clinic appointments and related stress if any	No large, randomized studies having evaluated or showed a higher benefit in treatment adjustment based on wearable sensors outputs if compared to standard of care (only data on one blinded not randomized trial on PKG suggested its additional value as add-on tool for treatment management)
+/− A support to remember pills intake	Not known how long any benefit of the devices lasted once they were not used any more
+/− A support to face the increment of PD prevalence against the lack of neurologists and the low rate of neurological visit/year	Is the accuracy the best outcome to evaluate a wearable sensor performance or rather its impact on PD symptoms/management and QoL?
+/− If a benefit on motor symptoms management will be strongly demonstrate (still not reached) this could impact on motor symptoms complications (reductions of falls, hip fractures and hospitalisation rate)	Not clear impact on QoL
	Not clear cost effectiveness profile

+/− indicates possible benefit, still not proven. *HY* Hoehn and Yahr.

### PDMonitor^®^

In Europe, it’s a CE marked, class IIa medical device.

### PKG^®^

The PKG System is the first FDA-cleared medical device on August 2014. It has received regulatory approval for the use in PD patients in Australia, Europe (CE marked) and US. It is a class IIa medical device.

### Sense 4 care—STAT-ON^®^

STAT-ON is a CE marketed, class IIa medical device.

### Kinesia™ technology

It is manufactured by Great Lakes NeuroTechnologies (Cleveland, Ohio, USA). It is FDA and CE marketed, class I medical device. However it is not marketed in several european countries.

### McRoberts—DinaPort

Is a CE marketed and FDA approved, Class I medical device in Europe and Class II in US.

### FeetMe^®^ monitor insoles

FeetMe® Evaluation is CE-marked class Im and FDA listed (510k exempt). It is manufactured by FeetMe, Paris, France.

### APDM™

It is FDA and CE marketed, class II medical device. Three different kits of Opal™ sensors can be bought, from the basic form to the most high-level system.

## Wearables sensors and non-motor Parkinsonian clinical features

The focus of wearable sensors is now also slowly shifting towards the broad, but more covert spectrum of NMS. Indeed, NMS are major determinants of quality of life, and influence the therapeutic strategy^119^. Few studies have addressed the question of the objective monitoring of NMS either using wearable sensors already available for motor features^120^, or using other devices such as smartwatches^121^. Therefore, the need for accurate and objective assessment of NMS in PD remains unmet.

Associations between NMS and the measure of bradykinesia and dyskinesia based on PKG have previously been reported^40^, particularly for sleep^33,53^ and impulse control disorders (ICDs)^122^. Regarding sleep, PKG parameters seem to correlate with different aspects of sleep including insomnia, parasomnia and restless legs syndrome^53^. However, PTI could not discriminate sleepy from motionless but non-sleepy PD patients and results on excessive daytime sleepiness are still inconsistent^123^. Yet, among patients complaining of excessive diurnal sleepiness, a small study has recently shown that PKG bradykinesia scores allow to distinguish between normal and abnormal PSG studies with good selectivity (86%) and sensitivity (80%), suggesting that this system is promising as a quantitative score for assessing sleepiness in PD^53^. Smartwatch sensors have also been proposed to quantify sleep stages in PD^121^, and to detect rem behaviour disorders, yet such device is not able to detect restless leg syndrome, or sleep apnea.

Psycho-behavioural manifestations of PD, such as ICDs and mood fluctuations, can directly influence therapeutic strategy and may vary throughout the day. However, unlike motor features, these manifestations are difficult to measure directly using remote digital technologies. One study suggested the possibility to indirectly measure dopamine-dysregulation syndrome (DDS) severity by measuring the amount of time a patient acknowledge treatment intake (consumption of medication) by pressing a button embedded in the PKG wearable device^122^. A high Response Ratio is reported as sensitive measure of impulsive compulsive behaviour^122^. Further digital technology developments are underway to capture psycho-behavioral disorders, such as tracking devices developed in the field of psychiatry to capture the dynamic nature of mood disorders using electronic self-report, behavioural data (collected through smartphone use: call logs, social media usage) and physiological measures^124^. Electronical ecological momentary assessment (EMA) appears to be a valuable tool for research on mood disorders, and involves the repeated administration of questionnaires requiring an immediate response^125^. Whereas questionnaires were previously administrated through paper surveys, wearable devices now allow to administer questionnaire repeatedly in a more instantaneous and convenient way at multiple time points along day. Chrono-record^126^ is one of these devices widely used in bipolar disorders research, and requires patients to rate their mood on a visual analogue scale ranging from 0 to 100. Patients can also add features of their mood state (reduced sleep, grandiose thoughts…). Thus, mobile apps and programs designed to record electronic self-report mood ratings are becoming common. However, such devices have limitations and remain subject to patient self-report, whereas clinical interviews are based both on verbal reports and clinical observations. To overcome these limitations, several studies have assessed smartphones sensors to monitor real-time behavioral patterns. Data extracted form phone usage activity including call and sms logs, and data from online social networks and reported usage patterns or language choices correlated with mood changes.

Detection of orthostatic hypotension can also impact PD management, and wearable blood pressure devices are capable of reproducing the results of standard blood pressure measurement in the supine and upright position with the advantage of a home environment^127^, but the main limitation being the size of the monitor. Smart watches can measure heart rate and possibly blood pressure and oxygen saturation, but only heart rate meets accuracy guidelines as of yet^128^.

Altogether, it seems that it will be soon possible to reliably measure several NMS, including orthostatic hypotension, sleep and excessive daytime sleepiness, using either devices worn on the body, smartphones or sensors embedded into a patient’s home (such as smart beds). To date, only one study suggested the possibility to screen DDSs^122^, using PKG, but does not allow to monitor those psycho-behavioral fluctuations. Other digital technology developments are underway to allow for better capture of often under-reported and under-recognised NMS, and allowing a comprehensive management of PD^129^. For example, PD_Manager is a mobile Health platform for PD patient’s management that will use a holistic approach combining the assessment of PD motor manifestations but also various NMS including sleep and mood^130,131^.

## Perspectives and conclusions

A more objective and continuous monitoring of PD features is an unmet need, related to both the difficulties in properly evaluating the presence and severity of symptoms by means of sole subjective means and by the fails in care continuity due to sparse in-person visits. Currently, patients have to purchase them at their own expense or neurologists can get recordings with various BWS throughout home healthcare providers. Although several BWS are available, there are no national or international guidelines on their scope of use and how to use them.

Our practical anthology aims to offer an european overview of BWS, aiming to improve PD patients management.

To conclude we propose a list of questions, which is not intended to represent formal guidelines, but rather reflect an authoritary opinion from european movement disorders experts with a variable field experience in BWS applications for PD patients. A summary of potential values and unknonw aspects/risks from BWS use is also proposed on Table 2.

1. Are BWS useful in monitoring PD motor and NMS manifestations? BWS could be useful in monitoring several PD motor features, including gait parameters, dyskinesia, motor fluctuations and tremor (see Fig. 1 and Table 1). Yet FoG and falls are detected only by few of them with variable levels of sensitivity/specificity. The interpretation of BWS data ideally requires a coupled analysis of objective measure (BWS) and patients’perspective or activities. Regarding NMS, a few evidence indicate the possibility to monitor orthostatic hypotension, sleep and excessive daytime sleepiness and DDS (with indirects measures) but this still needs confirmation.
2. Are BWS useful in the management of PD motor and NMS? We still do not know. There is some evidence that suggests a moderate/high correlation between changes in PKG scores (for bradykinesia, dyskinesia, and tremor) and Kinesia measure and treatment modifications^31,132^. However, studies investigating the effect of treatment modifications based on BWS alone vs. clinician-based decision alone are lacking. At this stage, we could only consider BWS as a potential useful add-tool for PD treatment management, when coupled with clinical evaluation (history taking) but their clinical relevance remains to be proven. Concerning NMS, there is a need for a trial that could indicate an impact of BWS on NMS management, even if the monitoring of a few NMS seem to be feasible.
3. Which BWS is most useful for which clinical feature? No direct comparison of these different tools has been performed in the same patients. Moreover not all BWS are intended to monitor the same PD manifestations and their performance may vary across clinical parameters.
4. Which BWS is most useful for which patients? As expressed in the previous question, there is no study that compared the accuracy of different BWS or the performance of one BWS in different PD groups in one study. Overall, the use of any BWS in demented parkinsonian patients still seem challenging due to the need of patient’s compliance. It could be discussed if PKG has the best wearability, possibly applicable to patients with cognitive impairment, although no study has investigated this aspect, while a single study explored physical activity in demented PD patients by means of DinaPort. At the same time, the low informative level of a clinical interview with cognitively impaired patients, could be the exact reason why we would need the help of a BWS for managing the treatment those patients.
5. Who is the most suitable physician or allied healthcare professional user profile for BWS in PD managment? Movement disorder experts and neurologists trained on BWS use and outcomes interpretation, but also general practitioners or PD nurses with similar training could consider the use of BWS for routine care in some selected patients. Several studies show that both healthcare professionnals and patients are ready to use the technology^133,134^. At the same time formats, data and lengths of the above described BWS reports are quite different, some reporting each single day, others offering the possibility to have an average of the whole recorded period. Once again, there no study comparing the usability of those different reports between them and this should be investigated.
6. Are BWS cost-effective? It has not been systematically investigated across different european countries. A cost-effectinevess study has been conducted in UK with inconsistent results. Inded a cost-saving effect of £17,362 has been found for PKG^135^ but the NICE committee recently commented that it was probably “overestimated” and the proposed calculation not reproductible^11^.

As final remarks we could consider a few additional points. First, the potential value of BWS for rehabilitation strategies, intended as add-on for patients’ management should not be underestimated. For instance, Feet ME has elaborated “Feet Me rehab”, a solution for individualized homecare rehabilitation, under supervision of a physical activity professor aiming to improve gait and balance impairment.

Secondly, if we widen our look outside Europe, it should be certaintly noted that in 2022 an Apple Watch-based tracking of PD motor features (tremor, dyskinesia) and self-reported information, has received clearance from the FDA, opening the path for the diffusion of ease of use monitoring systems for parkinsonian manifestations^136^. Concomitantly, in the Netherland, a wrist- and a hip-worn commercial activity trackers (AT) has been recently compared to a research-grade Dynaport Movemonitor (DAM) among 28 PD patients and 30 healthy controls, obtaining a very high correlation (*R*^2^ = 0.90) for the number of steps/day, but a worse performance for motor fluctuations detections (overestimated with AT if compared to DAM), with no ability of these BWS to differentiate PD from controls regarding daily fluctuations^137^. Nevertheless, AT based measures will be likely more and more offered as “monitoring tools”, claiming the need to better evaluate their role for PD management.

Overall, trust in the physician is built step by step over successive discussion, especially about how to analyze the reported observations and complaints of the patient, to convert them into therapeutic optimization. This aspect is a key point in favour of the therapeutic alliance^138^. Neurologists and other health-professionals should carefully consider patients’ reactions and preferences for eliciting collaboration and treatment adherence, favoring a tailored patient-centred standard of care, and we believe this model should be kept when using complementary information from BWS. Despite the current uncertainties, we should move toward progress and develop collaborative efforts to clarify the optimal scope and methods of BWS use in routine care of PD patients.
